# Increasing the efficacy of radiotherapy by modulating the CCR2/CCR5 chemokine axes

**DOI:** 10.18632/oncotarget.13287

**Published:** 2016-11-11

**Authors:** Kelli A. Connolly, Brian A. Belt, Nathania M. Figueroa, Aditi Murthy, Ankit Patel, Minsoo Kim, Edith M. Lord, David C. Linehan, Scott A. Gerber

**Affiliations:** ^1^ Department of Microbiology and Immunology, University of Rochester Medical Center, Rochester, NY 14642, USA; ^2^ Department of Surgery, University of Rochester Medical Center, Rochester, NY 14642, USA

**Keywords:** radiotherapy, myeloid cells, chemokines, immunotherapy, immune response

## Abstract

Although radiotherapy (RT) is widely used to control tumor growth across many cancer types, there is a relatively high incidence of RT failure exhibited by tumor recurrence, therefore a clear need exists to achieve improved effectiveness of RT. The RT-elicited immune response largely impacts the efficacy of RT and includes immune cells that kill tumor cells, but also immunosuppressive cells, which dampen anti-tumor immunity. Using murine models in which syngeneic tumor cell lines (Colon38, Glioma261, Line1) are grown intramuscularly and treated with 15 Gy local RT, we assessed the effects of RT on both the systemic and intratumoral immune response. Here we demonstrate that RT stimulates increased production of two chemokines, CCL2 and CCL5, at the tumor site. Further, that this leads to increased CCR2+ CCR5+ monocytes in circulation and subsequently alters the intratumoral immune infiltrate favoring the largely immunosuppressive CCR2+ CCR5+ monocytes. Importantly, a CCR2/CCR5 antagonist administered daily (15 mg/kg subcutaneously) starting two days prior to RT reduces both circulating and intratumoral monocytes resulting in increased efficacy of RT in radioresponsive tumors. Overall, these data have important implications for the mechanism of RT and present a means to improve RT efficacy across many cancer types.

## INTRODUCTION

Radiotherapy (RT), a benchmark therapy commonly administered across many types of cancers, relies in part on the magnitude of the induced immune response [1–3]. The immune response generated can be thought of as a double-edged sword, as RT can stimulate protective anti-tumor immune cells, however this therapy can also induce an immunosuppressive response that ultimately dampens the efficacy of RT [4, 5]. Myeloid cells, including monocyte precursors, often make up a large portion of the intratumoral immune infiltrate, and have been largely attributed with suppressing this anti-tumor immune response [6]. To this end, preclinical studies that broadly target the intratumoral myeloid cells have augmented the anti-tumor responses in some RT models [4, 7–10]. Furthermore, it has been recently established that high preoperative circulating monocyte levels negatively correlate with prognosis across many types of solid tumor malignancies [6, 11–15]. These findings have led to successful clinical trials in which therapies that block the infiltration of inflammatory monocytes (IM) into tumors have shown unanticipated promise [16]. However, the impact of radiotherapy on the intratumoral infiltration of this cell population remains largely unexplored. In this report we aim to fill this critical gap in knowledge and to utilize the information gained to improve the efficacy of radiotherapy.

Monocytes, a precursor to several suppressive myeloid cell types, are important immune-regulators that play a role in tissue repair and homeostasis [17–19]. Recruited to the site of infection or injury, these cells can be divided into two main populations: classical proinflammatory monocytes, and non-classical patrolling monocytes [17]. In mice, the expression levels of CCR2, a chemokine receptor, and Ly6C, a cell surface protein, are the primary distinguishing features of these two cell populations. Classical monocytes, or inflammatory monocytes (IM), express high levels of both CCR2 and Ly6C on their surface, whereas non-classical monocytes have low to absent expression levels of these molecules [17]. Inflammatory monocytes are recruited to tissues during inflammation, in part due to the recognition of CCR2 ligands present at the site of insult [20]. Once IM infiltrate tissues they often differentiate into macrophages or dendritic cells that exhibit either proinflammatory or anti-inflammatory characteristics based on the cytokine milieu [18]. Importantly, in tumors, these cells often differentiate into tumor-associated macrophages (TAMs), which suppress the anti-tumor immune response and promote the progression of cancer [21–23].

Tumor derived factors have been shown to actively recruit immunosuppressive IM into solid malignancies. Importantly, several myeloid cell chemoattractants are increased across different tumor tissues including the predominate ligand for CCR2+ IM: CCL2. Additional chemokine receptors expressed by IM, such as CCR5, have also been implicated in the promotion of intratumoral myeloid cell infiltration. Furthermore, the ligands for these receptors (CCL2 and CCL5) are often produced at increased levels by tumor tissues [24, 25] and, as a result, these ligands and their cognate receptors on IM have been targeted as a means to reduce tumor growth [6, 16, 26–33]. Schmall *et. al*. demonstrated that treating mice with a CCR2 antagonist led to reduced tumor growth and fewer metastases in the lung [27]. Similar results have been observed in mouse models of breast and pancreatic cancers [6, 26, 30], which have led to several successful phase 1b clinical trials utilizing a small molecule CCR2 inhibitor in late stage pancreatic patients [16]. Additional studies performed by Halama *et. al*. targeted CCR5 in patients with advanced stage colorectal cancer, and demonstrated effective reprogramming of the intratumoral myeloid cells that promoted, rather than suppressed, the antitumor immune response [28]. This work demonstrates that although the chemokine crosstalk in the tumor microenvironment (TME) is a complex and overall poorly understood process, both the CCL2:CCR2 and CCL5:CCR5 ligand/receptor pairs have been identified as likely therapeutic targets across several types of cancers.

Here, we determine that both the CCL2:CCR2 and CCL5:CCR5 chemokine axes are uniquely modulated by RT in various tumor models. The radiation-induced increase in the production of these chemokines results in the infiltration of a higher number of potentially protumorigenic CCR2+ CCR5+ IM both intratumorally and in circulation. Further, targeting this cell population using a dual antagonist of CCL2 and CCL5 improves the efficacy of RT overall.

## RESULTS

### Radiotherapy exacerbates the increase of circulating inflammatory monocytes observed in tumor bearing mice

**Figure 1:**
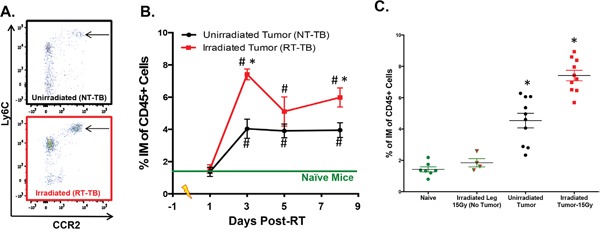
Radiotherapy increases peripheral blood IM 1x10^5^ Colon38 tumor cells were injected i.m. in C57BL/6 mice and left untreated or treated with 15 Gy radiation on day 7 of tumor growth (equivalent to day 0 on Figure 1 x-axis). **A**. Representative flow cytometry plots illustrating peripheral blood CCR2+ IM (arrow) from unirradiated and day 3-post RT treated mice. Plots are gated on CD45+, CD11b+ cells, and IM were shown to be Ly6G negative. **B**. The percentage of IM of CD45+ cells in the peripheral blood at various timepoints post-RT was determined by flow cytometry. In **C**. peripheral blood IM from naïve non-tumor-bearing mice + local RT (15 Gy) to leg, tumor-bearing, and tumor bearing + local RT (15 Gy) were plotted from the day 3 post-RT (day 10 of tumor growth) timepoint. # (p < 0.05) represents significance relative to naïve group and * represents significance to unirradiated tumor group as determined by ANOVA followed by a Tukey post-hoc test. n=4-10 for all groups at each time point.

It has been observed that levels of circulating inflammatory monocytes (IM) negatively correlate with prognosis across several types of cancers [6, 11–15, 34–36]. However, it is unclear how common cancer modalities such as radiotherapy (RT) impact the number of circulating IM. To address this, we studied the radiation response in a murine model of colon adenocarcinoma, Colon38, where 1x10^5^ tumor cells were injected intramuscularly into syngeneic C57BL/6 mice and established tumors were treated locally with 15 Gy RT 7 days after inoculation. Peripheral blood was collected from RT-treated tumor-bearing (RT-TB) mice and non-RT-treated tumor-bearing (NT-TB) mice and the number of circulating IM (CD45+, CD11b+, Ly6C(high), Ly6G-, CCR2+) was determined by multicolor flow cytometry. Day 3 post-RT representative dot plots illustrate that circulating IM (black arrow) are predominately CCR2+, and more importantly, are increased in RT-TB when compared to NT-TB mice (Figure 1A). We performed a kinetic analysis (1, 3, 5, and 8 days after RT) comparing the percentage of circulating IM among naïve, NT-TB and RT-TB mice. Consistent with observations from the literature, NT-TB mice exhibited an increase of circulating IM when compared to naïve control mice (Figure 1B). Although the levels of IM in the blood were similar in both groups one day after RT, the number of IM was significantly higher in RT-TB mice compared to NT-TB mice 3 days post RT. These data suggest that there is a delayed response to local RT that can be identified systemically. Intriguingly, five days after RT the levels of IM in the blood of RT-TB mice increased in variability and were not significantly different from levels in NT-TB mice, however IM levels in RT-TB mice were again significantly increased compared to NT-TB mice eight days post-RT. From these data we postulate that a large portion of IM could be leaving the bloodstream and migrating into tumor tissue approximately 5 days post-RT.

To determine whether the striking increase of IM observed 3 days post-RT was a response to normal tissue damage caused by RT, we performed a similar experiment with non-tumor bearing mice with or without administering 15 Gy radiation to the leg. Three days after RT, we collected peripheral blood from non-RT-treated non-tumor-bearing mice (naïve controls) and RT-treated non-tumor bearing mice, and compared the number of peripheral blood IM to NT-TB and RT-TB mice at the same time point. As expected, the presence of an established tumor caused a systemic increase in circulating IM compared to non-tumor-bearing controls and the number of IM was further enhanced in RT-TB mice (Figure 1C). Importantly, the number of circulating IMs from non-tumor-bearing mice treated with 15 Gy RT to the leg was not increased compared to naive control mice at this time point. This suggests that the increase in IM is a response of the tumor to RT rather than a response of normal tissue to damage caused by RT.

### Radiotherapy increases the number and proportion of intratumoral inflammatory monocytes

**Figure 2:**
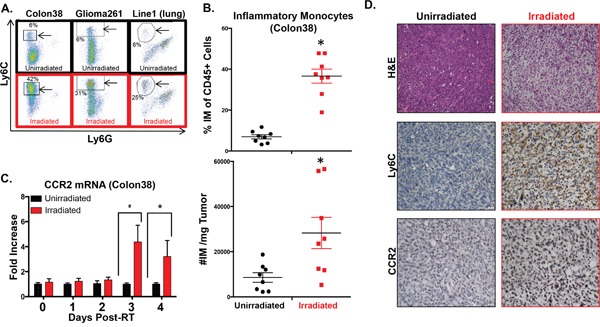
Intratumoral IM are increased following RT Colon38, Glioma261, and Line1 tumor cells were injected and irradiated as described in Figure 1 and the materials and methods. **A**. Representative dot plots of intratumoral IM (arrowed black box/circle) assessed by flow cytometry from day 4 post-RT (day 11 of tumor growth) unirradiated and irradiated tumors. Percentage of IM out of CD45+ cells are provided on plots. IM from Colon38 tumors were quantified by %IM of total CD45+ cells (**B**- top) and these data were normalized based on tumor size and shown as #IM/mg tumor (B- bottom). **C**. mRNA was isolated from Colon38 tumor homogenate and CCR2 expression was determined by RTPCR at various timepoints post-RT. **D**. Immunohistochemistry was performed on day 11 unirradiated and irradiated (4 days post-IR) Colon38 tumors as described in materials and methods. * (p < 0.05) represents significance as determined by t-test. n=4-8 for all groups at each time point.

We next determined if CCR2+ IM were present intratumorally and whether RT modulated the number of these cells. We performed an experiment examining the percentage of intratumoral IM in unirradiated to irradiated tumors at day 4 post-RT, which is one day after the observed spike of circulating IM. Colon38 tumors were harvested from mice, processed into single cell suspensions, and stained with fluorescent antibodies to assess the total immune infiltrate of each tumor by flow cytometry. Representative flow cytometry dot plots from unirradiated (black; top) and irradiated (red; bottom) tumors demonstrate a dramatic increase of IM in RT-treated tumors (Figure 2A). Proportionally to all immune cells, IM were significantly increased by approximately 4-fold in RT-treated tumors at this time point and similar data were obtained when the number of IM was normalized for tumor size (Figure 2B). As expected, the majority of intratumoral IM (CD45+, CD11b+, Ly6C(high), Ly6G-) were positive for CCR2 expression and represented the vast majority of all CCR2+ intratumoral cells (data not shown). Using a similar radiation/tumor model as Colon38, we observed increases in intratumoral IM following RT in a model of glioblastoma, (Glioma261-syngeneic with C57BL/6 mice) as well as in a model of lung carcinoma (Line1-syngeneic with BALB/c mice) suggesting that this phenomenon is generalizable across cancer types and strains of mice (Figure 2A). Focusing on the Colon38 model, we demonstrated that CCR2 mRNA expression was unchanged between RT-treated and untreated tumors at early time points, however they were significantly elevated in RT-treated tumors relative to non-RT-treated tumors three and four days post-RT (Figure 2C). These data suggest that the influx of intratumoral CCR2+ IM does not occur immediately following RT, but is delayed and occurs *after* the increase of circulating IM (Figure 1). Immunohistochemical analysis of the tumors (day 4 post-RT) revealed striking changes to irradiated tumors when compared to unirradiated tumors. For example, RT decreased the density of tumor cells while increasing the infiltration of immune cells as assessed by hematoxylin and eosin staining (Figure 2D-top images) and CD45+ staining (data not shown) respectively. Importantly, the level of Ly6C+ (Figure 2D-middle images) and CCR2+ (Figure 2D-bottom images) cells (surface markers predominately found on IM), were greatly increased and uniformly distributed in irradiated tumors. These complementary data demonstrate that RT results in an altered intratumoral immune infiltrate characterized by a striking increase of CCR2+ IM three-four days post-RT.

### Radiotherapy results in the induction of chemokines that promote migration of myeloid cells

**Figure 3:**
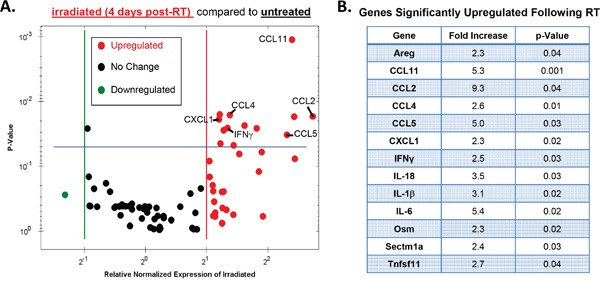
Radiotherapy modulates various intratumoral cytokines and chemokines Tumors were injected and irradiated as outlined in Figure 1, and mRNA from day 4 post-RT (day 11 of tumor growth) tumor homogenate was used to examine the expression of various cytokine and chemokines using an BioRad RTPCR plate array. **A**. A volcano plot illustrating changes in gene expression between irradiated and unirradiated tumors where red defines upregulated genes (2-fold induction), black defines no change, and green defines downregulated genes. Genes that exhibited a significant increase in expression in irradiated tumors (p<0.05) fall above the blue line and are listed in **B**. Significance determined by t-test. n=3 for each group.

To gain a more comprehensive assessment of the impact that RT has on the inflammatory milieu of the tumor microenvironment, we used RTPCR to measure the expression of various cytokine/chemokine genes 4 days post-RT (Figure 3). A complete list of genes contained in the RTPCR plate with levels of regulation and p-values can be found in the Supplementary material (Supplementary Table S1). These data are presented as a volcano plot comparing RT-treated to non-RT-treated tumors where down-regulated genes are shown as green, unchanged as black, and up-regulated as red (Figure 3A). No genes assessed were significantly downregulated whereas 13 genes were significantly up-regulated (p≤0.05) in RT-treated compared with non-RT-treated tumors (Figure 3A-3B).

As expected, the proinflammatory cytokines IFNγ, IL-18, and IL-1β were significantly upregulated in RT-treated tumors as these genes have been previously associated with the antitumor response elicited by RT [1, 3, 37, 38]. Other genes found to be significantly upregulated include several growth factors (Areg, Osm), and one anti-inflammatory cytokine (IL-6). Notably, all but one of the significantly upregulated chemokine genes have been shown to act as ligands for the IM-expressed chemokine receptors CCR2 (CCL2, CCL11) and/or CCR5 (CCL11, CCL4, CCL5) [39]. These data suggest that RT results in an intratumoral induction of chemokines known to be responsible for the chemotaxis of myeloid cells. Taken together, our results illustrate a complex immunological response to RT in the tumor microenvironment that consists of antitumorigenic as well as protumorigenic characteristics. From the array data we identified two chemokine ligands, CCL2 and CCL5, which have been shown to be primarily responsible for mediating IM migration [40], therefore we focused the remainder of our studies on assessing how RT modulates these chemokine transcripts.

### Intratumoral CCL2 and CCL5 are increased by radiotherapy

**Figure 4:**
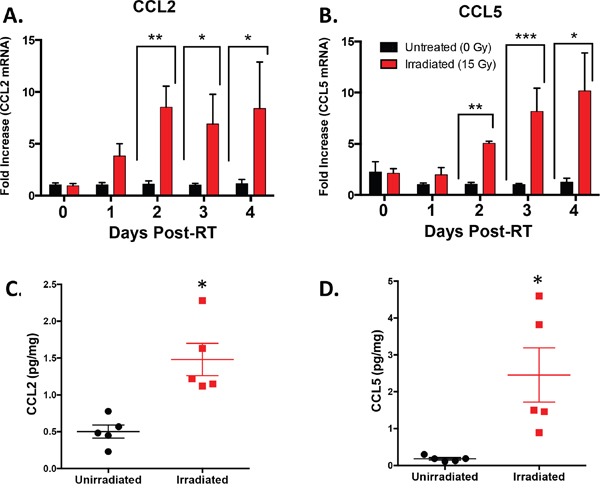
Intratumoral CCL2 and CCL5 are induced by RT Tumors were injected and irradiated as described in Figure 1. mRNA was isolated from tumor homogenate and CCL2 **A**. and CCL5 **B**. expression was determined by RTPCR at various timepoints post-RT. CCL2 **C**. and CCL5 **D**. protein expression was quantified by ELISA from day 4 post-RT (day 11 of tumor growth) tumor homogenate. * (p < 0.05) represents significance by t-test. n=4-5 for all groups at each time point.

To investigate the kinetics of CCL2 and CCL5 production in the tumor microenvironment, we used the same Colon38 tumor/RT model as before and harvested tumor tissue at different timepoints post-RT, isolated RNA, and quantified the relative amounts of CCL2 and CCL5 transcript by qRT-PCR. We determined that CCL2 transcript levels in irradiated tumors were increased up to ten fold when compared to unirradiated tumor controls 2, 3, and 4 days post-RT (Figure 4A). Additionally, CCL5 was induced in RT-treated tumors with similar kinetics (Figure 4B).

To verify that increased transcript levels coincide with increased CCL2 and CCL5 protein levels, we harvested unirradiated and irradiated Colon38 tumors on day 11 (four days post-RT) and measured protein levels by ELISA (Figure 4C & 4D). We determined that irradiated tumors had significantly increased levels of both CCL2 and CCL5 protein compared to unirradiated control tumors at this time point. Overall, these data indicate that RT increases the production of the monocyte chemokines CCL2 and CCL5 at both the transcript and protein levels *in vivo*.

We examined if RT modulated the surface expression of CCR2 and CCR5 on IM. Although the cell surface expression level of CCR2 did not change on intratumoral IM from irradiated and non-irradiated tumors (data not shown), we did observe an increase of CCR5 on the cell surface of IM from irradiated tumors when compared to non-irradiated tumors (Supplementary Figure S1). Since CCR5 supports the transendothelial migration of monocytes [41], the RT-induced increase of this chemokine receptor on IM, along with intratumoral increases of CCL2 and CCL5 (Figure 4), may facilitate the infiltration of these myeloid cells into irradiated tumors.

### Radiotherapy stimulates an upregulation of CCL2 and CCL5 transcripts in various tumor cell lines

To determine if RT stimulates tumor cells across different tumor models to produce the chemokines CCL2 and CCL5, we irradiated *in vitro* cultures of various murine and human tumor cell lines and measured CCL2 and CCL5 transcript levels by qRT-PCR (Table 1). Strikingly, we found that the majority of cell lines tested responded to radiation by either a transient or sustained upregulation of CCL2 and CCL5 transcript levels. A murine melanoma (B16) and a lung carcinoma cell line (LLC) appear to have delayed upregulation of CCL2 and CCL5 transcript levels. Additionally, the colon adenocarcinoma line used throughout this study (Colon38), a breast adenocarcinoma (E0771), and a second lung carcinoma cell line (Line 1) demonstrate transcript up-regulation that is either short-lived or sustained following RT. A similar response to RT was observed in three human tumor lines (MCF7, H460, & OVCAR-1). These results implicate the CCL2:CCR2 and CCL5:CCR5 axes in the cellular response to RT across a diverse array of cancer types, both human and murine.

**Table 1:** Irradiated (15 Gy) tumor cells express increased amounts of CCL2 and CCL5 mRNA when compared to unirradiated tumor cells *in vitro*

*Tumor Line*	Fold Increase Over Non-Irradiated Cells							
	CCL2				CCL5			
	*Day 1*	*Day 2*	*Day 3*	*Day 4*	*Day 1*	*Day 2*	*Day 3*	*Day 4*
**B16**	*.91*	*2.2*	*1.6*	*4.6*	*3.5*	*4*1	*115*	*164*
**Colon38**	*2*	*11*	*27*	*24*	*13*	*67*	*367*	*117*
**E0771**	*6.2*	*32*	*31*	*22*	*44*	*350*	*286*	*170*
**Glioma 261**	*.70*	*0.5*	*30*	*60*	*1.6*	*8.0*	*29*	*29*
**LLC**	*3.6*	*3.3*	*7.1*	*23*	*7.7*	*12*	*13*	*3.1*
**Line1**	*5.9*	*897*	*752*	*125*	*6.1*	*113*	*179*	*111*
**H460**	*.29*	*0.35*	*1.1*	*3.5*	*2.6*	*8.0*	*8.8*	*5.8*
**MCF7**	*NE*	*NE*	*NE*	*NE*	*1.8*	*1.8*	*6.1*	*6.2*
**OVCAR1**	*2.3*	*5.5*	*7.6*	*34*	*2.1*	*3*	*8*	*24*

Various tumor cell lines were plated *in vitro* and initial cell density was adjusted to ensure that a similar number of cells were analyzed at each endpoint. Tumor cells were irradiated with 15 Gy, cultured, washed to remove dead cells, and RNA isolated and probed for CCL2 and CCL5 by RT-PCR at various time points. Data is normalized to GAPDH and expressed as fold increase over non-irradiated cells from that time point.

NE = Not Expressed.

### Dual CCR2/CCR5 blockade (CVC) improves efficacy of RT in radioresponsive tumors by specifically targeting IM

**Figure 5:**
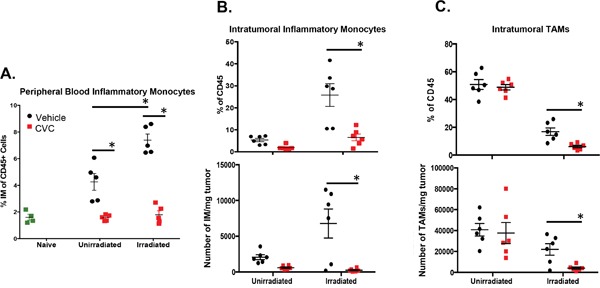
A CCR2/CCR5 small molecule antagonist (CVC) reverses the radiation-induced increase of IM in peripheral blood and tumor Tumors were injected and irradiated as described in Figure 1. Starting two days prior to irradiation, mice were treated daily with 15 mg/kg drug or vehicle control s.c. for 15 days. **A**. Peripheral blood was isolated from the different groups of mice on day 10 (day 3 post-RT) to assess levels of IM by flow cytometry. Day 11 (day 4 post-RT) tumors were dissociated and intratumoral IM **B**. or TAMs **C**. quantified by flow cytometry by percentage of total CD45+ immune cells (top panels) and by number of cell/mg tumor tissue (bottom panels). * (p < 0.05) represents significance as determined by t-test. n=4-6 for all groups.

Having identified that RT facilitates the infiltration of IM, a potentially tumor-promoting cell type, into the tumor microenvironment, we next determined whether blocking this infiltration could improve the efficacy of RT. We used our previously described mouse model and treated mice with 15 mg/kg of a small molecule dual inhibitor against CCR2/CCR5 (CVC) daily starting 2 days before RT. CVC was effective in reducing the numbers of circulating IM in both unirradiated and irradiated tumor-bearing mice to the levels of non-tumor-bearing control mice (Figure 5A). CVC also resulted in a significant decrease in the proportion and number of both IM (Figure 5B) and typically immunosuppressive TAMs (predominantly derived from differentiated IM) (Figure 5C) in irradiated tumors compared to vehicle controls. These results suggest that CVC is hitting the intended target (IM) both in the peripheral circulation as well as in the tumor.

**Figure 6:**
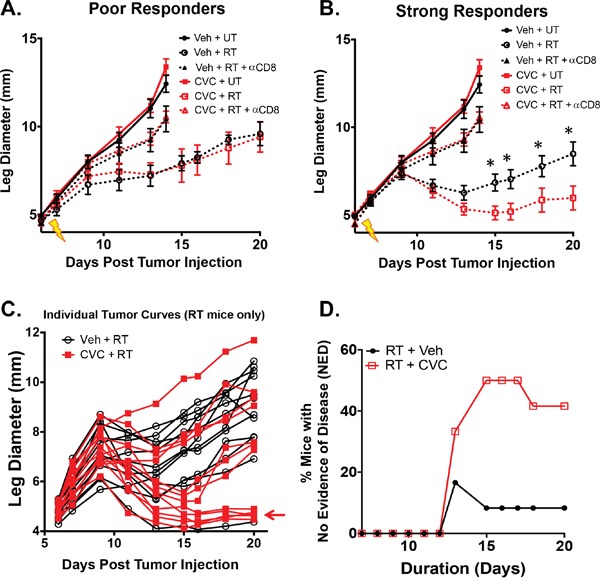
Dual blockade of CCR2/CCR5 (CVC) improves RT efficacy in radioresponsive tumors Tumors were injected and irradiated as described in Figure 1. Starting two days prior to irradiation, mice were treated daily with 15 mg/kg CVC (CCR2/CCR5 antagonist) or vehicle control s.c. for 15 days. Tumor growth was monitored in unirradiated along with poorly radioresponsive **A**. and strongly radioresponsive **B**. tumors. The same experiment was performed in conjunction with CD8+ T cell depletion (200 ug anti-CD8 given every 3 days starting on day 4). Untreated and anti-CD8 data from (A) is duplicated in (B) for reference. Individual growth curves (poor and strong responders combined) from both irradiated vehicle (black) and CVC (red) treated mice are presented in **C, D**. Percent mice with no evidence of disease (NED) was calculated when tumor size was reduced to non-tumor leg measurements between the irradiated +/- drug groups. * (p < 0.05) represents significance as determined by ANOVA followed by a Tukey post-hoc test. n=8 for both unirradiated groups; 3-4 for poor responders; 8-9 for strong responders. n=6 for anti-CD8 studies. n=12 for NED plot.

To determine if dual blockade of these chemokines enhances the efficacy of RT, tumor growth was monitored in irradiated and unirradiated tumors with or without CCR2/CCR5 inhibitor. CVC had no significant effect on growth in non-RT-treated tumors (Figure 6A). Based on our previous data in this model we know that radiotherapy of Colon38 tumors results in strongly radioresponsive and poorly radioresponsive tumors, which can be determined based on the change in tumor size from 2 to 4 days after RT as previously described [2]. Tumors that increase in size during this time period are considered to be “poor responders” whereas tumors that decrease in size during this time period are considered “strong responders”. CCR2/CCR5 inhibition in irradiated tumors did not change the ratio of strong responders to poor responders (data not shown). Interestingly, CCR2/CCR5 inhibition enhanced the efficacy of RT but only in strongly radioresponsive tumors. For example, CVC did not enhance the effectiveness of RT in *poor responders* as vehicle and CVC-treated irradiated tumors did not differ in size (Figure 6A). Intriguingly, CVC significantly improved the efficacy of RT in *strong responders* as the irradiated tumors of CVC-treated mice were significantly decreased in size compared to tumors from vehicle-treated mice 8 days after RT and this difference persisted until drug treatment was stopped 13 days post-RT (Figure 6B). Individual growth curves of both RT + vehicle and RT + CVC mice are presented in Figure 6C and illustrate more tumors showing a reduction of tumor burden particularly in the CVC group (see arrow). Also striking was the number of mice with no evidence of disease (NED) (tumor size is <5 mm, which represents the normal leg diameter) in the CVC-treated irradiated tumor group compared to the vehicle-treated irradiated tumor group (Figure 6D). Thirteen days post-RT, 40% of all CVC-treated irradiated tumors exhibited NED compared to 10% of vehicle-treated irradiated tumors. Notably, this increase NED was observed when both strong responders and poor responders to RT were included together. These data indicate that dual blockade of CCR2/CCR5 increases the efficacy of RT in radioresponsive tumors and that the benefits of combination therapy are maintained throughout the treatment period.

We previously demonstrated that CD8+ T cells were required to mediate the anti-tumor effects of RT [1–3] especially in radioresponsive Colon38 tumors [2]. We assessed whether CVC was acting independently or in concert with CD8+ T cells to enhance the efficacy of RT in strong responders. Using a similar protocol as described for Figure 5/6, we antibody-depleted mice of CD8+ T cells and assessed tumor growth following RT in conjunction with CVC or vehicle treatment. As expected, CD8+ T cell depletion reduced the anti-tumor effects of RT (Figure 6A & 6B-triangles), but more importantly, it rendered CVC therapy completely ineffective on enhancing the efficacy of RT as tumor burden was identical between CVC and vehicle treated mice in the absence of CD8+ T cells (Figure 6B-triangles). These data confirm that CD8+ T cells are indispensible in this RT model even in the presence of dual CCR2/CCR5 inhibition, and suggest that CVC therapy is likely amplifying an existing radiation-induced anti-tumor T cell response.

## DISCUSSION

It is well established that RT elicits an antitumor immune response that is critical to treatment efficacy [1, 2, 42–44]. There are many reports describing the RT-elicited anti-tumor contribution from the immune system. However, in this manuscript we identify an RT-induced mechanism that may counteract the many positive benefits induced by this cancer treatment modality. These findings are supported clinically as RT, although a widely used cancer treatment, is often insufficient to achieve cures on its own [45–48]. Therefore, we sought to improve the efficacy of RT by understanding the complex immunological response that it elicits. Our data demonstrate that the antitumor immune response is quickly dampened via an immuno-regulatory program commandeered by the tumor microenvironment (TME) and that RT initiates this suppressive response.

In support of this idea, we have shown that the intratumoral myeloid cell infiltrate following RT is skewed towards an increased population of IM, a cell type previously reported as being immunosuppressive [6, 22, 23]. Moreover, this myeloid infiltrate is preceded by intratumoral increases in the levels of CCL2 and CCL5, both shown to be monocyte chemoattractants [6, 25, 30, 33, 40, 41]. Interestingly, we’ve demonstrated that RT rapidly, and with varying kinetics, increases the production of CCL2 and CCL5 across several different murine and human cancer cell lines *in vitro* (Table 1). These data, together with data from our cytokine/chemokine RTPCR analysis, suggest that irradiated cancer cells across different cancer types, from both murine and human origin, could be hijacking this immuno-regulatory program to recruit myeloid cells (such as IM) and promoting tumor progression. This data is supported by the work of Kozin *et. al*. who demonstrated an increase in stromal-derived factor 1a, a chemokine produced in the TME after local RT, that led to an increase in intratumoral myeloid cells and promoted tumor growth [5].

Importantly, the makeup of the intratumoral immune infiltrate varies based on tumor type with some containing mostly myeloid cells and others being primarily lymphocytic [49]. The types of immune cells that a certain TME draws is liable to have a large impact on the effectiveness of combining CCR2/CCR5 antagonism with RT. Tregs, which express CCR5 on their surface that facilitates migration into tumor tissues [32], elicit potent immunosuppression resulting in tumor progression [50]. Although the Colon38 tumor model has a minimal regulatory T cell (Treg) component (Supplementary Figure S2), CCR2/CCR5 antagonism in tumors with abundant Tregs could offer additional benefits by targeting Tregs as well as IM post-RT and promote anti-tumor immunity by blocking two modes of immunosuppression.

CCR2 and CCR5 have been shown in some cases to be expressed on activated T-cells and natural killer (NK) cells [51]. Therefore, blockade of these chemokines could abrogate entry of these important anti-tumor immune populations. However, our data demonstrate that intratumoral T-cells, along with NK cells, were not reduced following administration of CVC but instead they remained unchanged or slightly increased following drug treatment (Supplementary Figure S3). Although we saw only slight changes in the lymphocytic cell populations, the combination of CVC greatly altered the intratumoral myeloid landscape. For example, we observed a decrease in the percentage and number of tumor-associated macrophages (TAMs) (Figure 5C), likely a result of blocking entry of their precursors, IM. Furthermore, both RT and CVC altered the phenotype of the remaining TAMs (data not shown) suggesting that this therapy not only reduces infiltration of these cells into the tumor, but may also modulate phenotype and perhaps function. Interestingly, we observed a compensatory increase in Ly6C+, Ly6G+ granulocytic cells (data not shown). Importantly, our lab and others have reported suppressive characteristics associated with these granulocytic cells in other tumor models and have been successful in demonstrating improved tumor control when targeting this population (manuscript in review). Nevertheless, the mechanism of this compensatory increase in granulocytic cells is likely complex and the role of these cells is undefined in the radiation model described here. Regardless, these observations speak to the complexity of the RT-elicited immune infiltrate and the possibility that targeting multiple cell types may be required to optimally enhance RT efficacy across different cancers.

We have shown that dual CCR2/CCR5 antagonism results in a depletion of IM intratumorally in RT-treated tumors. However, it is unclear the exact mechanism(s) of how CVC is reducing the number of intratumoral IM. On one hand, CCR2/CCR5 antagonism is clearly preventing a tumor-associated increase of IM in the peripheral blood (Figure 5A), and since systemic numbers of IM are largely regulated by bone marrow output, it is possible that dual blockade of CCR2/CCR5 is preventing mobilization of IM from the bone marrow into the peripheral blood. On the other hand, migration of circulating monocytes across endothelium towards a chemokine gradient has been shown to be necessary for routine tissue surveillance [40] and entry into tumors [25]. Since RT increases intratumoral CCL2/CCL5 creating a positive chemoattractant gradient in the TME and facilitating migration of IM into the tumor, CVC may also interfere with the extravasation of IM from the blood stream into the tumor. It is likely that targeting both processes via CCR2/CCR5 inhibition blocks myeloid entry into the tumor resulting in improved RT efficacy of radio-responsive tumors.

Inhibiting the infiltration of CCR2+ CCR5+ IM by way of the CCL2:CCR2 and CCL5:CCR5 axes results in increased efficacy of RT. These data further support the notion that IM are suppressing the radiation-induced anti-tumor immune response. Interestingly, dual antagonism of CCR2 and CCR5 was only advantageous in *radioresponsive* tumors. Previously we have characterized a phenomenon in this tumor radiation model wherein tumors are treated identically but exhibit variable responses to RT [2], recapitulating a difference in RT efficacy often observed clinically. We have shown that approximately half of RT-treated tumors *respond strongly* to RT and decrease in size 2 to 4 days post-RT, whereas the rest continue to increase in size from 2 to 4 days following RT classifying them as *poor responders* [2]. Although the exact cause for the variation in response to RT is unknown, we demonstrated that there is a productive anti-tumor T-cell response in *strong responders* compared to *poor responders* [2]. For example, *strong responders* contain a higher proportion of CD8+ T-cells that have increased effector capabilities on a per-cell basis compared to those of *poor responders*. We hypothesize that the efficacy of CCR2/CCR5 dual blockade is dependent on the existence of a productive *initial* anti-tumor T-cell response.

In support of this hypothesis, we did not observe any effect of CVC in non-RT-treated tumors, tumors in mice that were depleted of CD8+ T cells, or *poor responders*. Based on our previous data, non-RT-treated and poorly radioresponsive tumors have an inherently diminished anti-tumor T cell response. Even though *poor responders* have received a strong inflammatory stimulus (RT), for reasons not yet known, the anti-tumor T-cells are not only reduced in number, but are also incapable of overcoming the suppressive mechanisms of the TME that are likely augmented by the influx of suppressive IM. However, strongly radio-responsive tumors demonstrate an increase in intratumoral IFNγ that may likely initiate a strong anti-tumor T cell response [2]. We postulate that depleting IM effectively sustains but does not generate the initial anti-tumor T-cell response. Therefore, this would suggest that there is reason to treat tumors, especially those with insufficient anti-tumor T-cell responses (*poor responders*), with a combination of therapies. We speculate that future studies involving the addition of immunotherapy aimed at promoting T cell effector functions, along with CCR2/CCR5 antagonism, would further enhance the efficacy of RT.

The dose of RT used throughout this manuscript (15 Gy) is considered an ablative radiation schedule. In our previous work in B16 melanoma we determined that this ablative dose was superior in controlling tumor burden when compared to a fractionated schedule [3]. Importantly, we also demonstrated that a greater anti-tumor immune response was generated by the ablative dose when compared to the fractionated schedule. Nonetheless, we examined if CVC could enhance RT efficacy in a clinically relevant fractionated RT schedule (5 Gy x 5) in the Colon38 model. Not surprisingly, administration of CVC to mice that received the fractionated schedule increased the efficacy of RT only slightly (data not shown). This result is likely due to the inferior anti-tumor immune response generated by the fractionated schedule compared to the ablative 15 Gy schedule. Therefore, CVC is likely to be most effective when combined with a RT schedule that generates a strong anti-tumor immune response.

Our results demonstrate that RT uniquely induces an increase of intratumoral IM and this concept may be generalizable across many cancers, as we have observed this phenomenon in multiple tumor models (Figure 2A). Additionally, CVC augmented the RT response in a radioresponsive group of tumors using a lung carcinoma model (manuscript in preparation). Overall, our findings are supported by a recent publication from Kalbasi *et. al*., showing an RT-specific increase in the production of CCL2 by tumor cells in pancreatic ductal adenocarcinoma models, and an increased efficacy of RT when this chemokine is depleted [52]. Interestingly, Kalbasi *et. al*. reports that the improved effectiveness of RT after CCL2 blockade is *not dependent* on CD8+ T-cells as therapy was still effective in the absence of CD8+ T cells. This is in contrast to our data where we find that CD8+ T-cells are essential to the efficacy of RT even in the presence of CVC (Figure 6). This disparity suggests that the mechanism of this novel immunotherapy could differ depending on the unique properties inherent to a variety of cancers. Nevertheless, even though the mechanism of action is different between reports, both clearly illustrate the benefit of targeting IM as a means to enhance RT.

Overall, our data indicate that circulating and intratumoral IM are increased in RT-treated tumor-bearing hosts as a result of a radiation specific up-regulation of the monocyte chemokine ligands CCL2 and CCL5. This is important, as the level of monocytes in preoperative cancer patients’ complete blood counts has been shown to correlate with poor prognosis across several tumor types [6, 11–15]. Due to the predominately restricted expression of CCR2 on IM in the blood, we suggest that CCR2+ IM could serve as a biomarker to identify candidates for CCR2/CCR5 blockade post-RT. Further, the surface expression of CCR2 on peripheral IM makes this cell population an attractive therapeutic target. Overall, the observation of an RT-specific recruitment of IM to the TME that promotes tumor growth has intriguing implications for our current understanding of the mechanism of RT efficacy.

## MATERIALS AND METHODS

### Tumor lines and mice

All cell lines were maintained in MAT/P medium (US patent 4.816.401) supplemented with 100 U/mL penicillin, 100 ug/mL streptomycin and 2% fetal calf serum (with the exception of Glioma 261 in 5% fetal calf serum). The cell lines, including murine lines syngeneic to C57BL/6 unless otherwise noted, used in these studies include Colon38 (from E. Brown, University of Rochester), a murine colon adenocarcinoma; B16 (from E. Lord, University of Rochester), a murine melanoma; E0771 (from E. Brown, University of Rochester), a murine breast carcinoma; Glioma 261 (from NCI), a murine glioblastoma; LLC (from NCI), a murine lung carcinoma; Line1 (from J. Yuhas, Oak Ridge National Laboratory), a murine lung carcinoma (syngeneic to BALB/c); OVCAR-1 (from C. Grossman, University of Rochester), a human ovarian carcinoma; H460 (from C. Grossman, University of Rochester), a human large cell lung carcinoma; MCF7 (from C. Grossman, University of Rochester), a human metastatic mammary adenocarcinoma. 6-8 week old female BALB/cJ and C57BL/6J mice were purchased from Jackson Laboratory (Bar Harbor, ME) and treated in accordance with the University Committee on Animal Resources’ approved guidelines.

### Tumor inoculation and treatment

A general tumor protocol was established where 1 x 10^5^ tumor cells were injected intramuscularly in the left legs of female C57BL/6J or BALB/cJ mice. Mice were treated locally with radiotherapy (RT) 7 days after tumor cell injection using a 3200 Curie-sealed ^137^Cesium source that operates at roughly 1.90 Gy/min. Jigs were constructed and designed to specifically treat the tumor-bearing leg with 15 Gy radiation [2]. This source and the collimators used are calibrated periodically to ensure equal distribution of radiation. Standard calipers were used to measure tumor growth as described previously [53]. Tumor-bearing mice were administered 15 mg/kg of a CCR2/CCR5 antagonist (named CVC, Tobira Therapeutics, CA) [54, 55] or vehicle control (40% Hydroxypropyl-beta-cyclodextrin [Acros Organics] & solutol HS15 [Sigma] in sterile water) subcutaneously (s.c.) 1X/day starting 2 days before RT for the indicated amount of time. CD8+ T cells were depleted by treating mice with 200 ug of anti-CD8 (clone 53-6.7) i.p. every 3 days beginning 4 days post-tumor inoculation. Rat IgG was used as a control in anti-CD8 experiments and did not affect tumor growth when compared to mice that did not receive rat IgG (data not shown).

### Flow cytometry

Peripheral blood was collected from tail veins at various time points into tubes containing heparin (Hospira, Inc.). Tumors were removed 4 days post-RT and processed into single cell suspensions as previously described [1]. A total of 1 x 10^6^ tumor cells and 15uL of whole blood were blocked with Fc Block (clone 2.4G2) followed by staining with a cocktail of directly conjugated primary antibodies (Supplementary Table S2) for 30 minutes. All samples were washed with 1 mL of PBS/1% BSA/0.1% azide, fixed with BD Cytofix/Cytoperm (BD Biosciences), and analyzed using a 12-color LSRII (BD Biosciences) and FlowJo software (Tree Star). Data is reported as percent of CD45+ events and normalized per milligram of tumor where indicated.

### Immunohistochemistry

All immunohistological stainings were performed on 5 μm sections cut from formalin-fixed, paraffin-embedded tissue. Gill's Hematoxylin #3 and Eosin Y (Polysciences Inc.) were used for H&E staining. Monoclonal antibodies used for immunohistology were as follows: Ly6C (clone ER-MP20) and CCR2 (clone E68) (Abcam). Tissue sections were subjected to a heat-induced antigen retrieval performed in 10 mmol/L citrate buffer (pH 6.0) in a pressure cooker and incubated with the primary antibodies followed by corresponding biotinylated goat anti-rat IgG and goat anti-rabbit IgG secondary antibody (Vector Laboratories). An Avidin/Biotin amplification kit (Vector Laboratories) and DAB detection kit (Dako) were used to reveal the positively stained cells with nuclei counterstained with hematoxylin. Images were taken using an Olympus DP80 microscope camera.

### CCL2, CCL5 protein quantification

Tumor homogenate was collected from RT-treated or non-RT-treated mice 4 days post-RT. We determined CCL2 and CCL5 concentrations by ELISA according to manufacturer's protocol (Peprotech). Values were normalized to total protein in homogenates as determined by Pierce BCA Protein Assay Kit (Thermo Scientific) and analyzed using a Synergy HTX Multi-Mode Reader (Biotek).

### qPCR based gene expression analyses

Total RNA was obtained using RNeasy Fibrous Tissue Mini Kit (Qiagen) and RNeasy Mini Kit (Qiagen) for tumor tissue and cell lines, respectively. RNA was transcribed into cDNA and quantitative real-time PCR (qRT-PCR) was conducted using predesigned SYBR Green Gene Expression Prime PCR Primers (GAPDH, qMmuCED0027497; CCL2, qMmuCED0048300; CCL5, qMmuCID0021047; CCR2, qMmuCED0049646; CCR5, qMmuCID0020341) and RTPCR Plates (BioRad) on a C1000 Touch Thermal Cycler (BioRad). Target gene expression was normalized to glyceraldehyde-3-phosphate dehydrogenase (GAPDH) and expressed as fold increase over control.

### Statistical analyses

All data were analyzed by one-way ANOVA, Tukey's test and/or student *t* test using GraphPad Prism version 6.0 (GraphPad Software Inc.), unless otherwise stated. *P* < 0.05 was considered statistically significant.

## SUPPLEMENTAL FIGURES AND TABLES


