# Supplementary material for: Increasing the efficacy of radiotherapy by modulating the CCR2/CCR5 chemokine axes
Source: Oncotarget. 2016 Nov 11;7(52):86522–35. doi: 10.18632/oncotarget.13287 (PMC5349932; doi:10.18632/oncotarget.13287)
Supplement: Supplementary file 1 [file oncotarget-07-86522-s001.pdf]

Increasing the efficacy of radiotherapy by modulating the CCR2/ CCR5 chemokine axes

SUPPLEMENTAL FIGURES AND TABLES

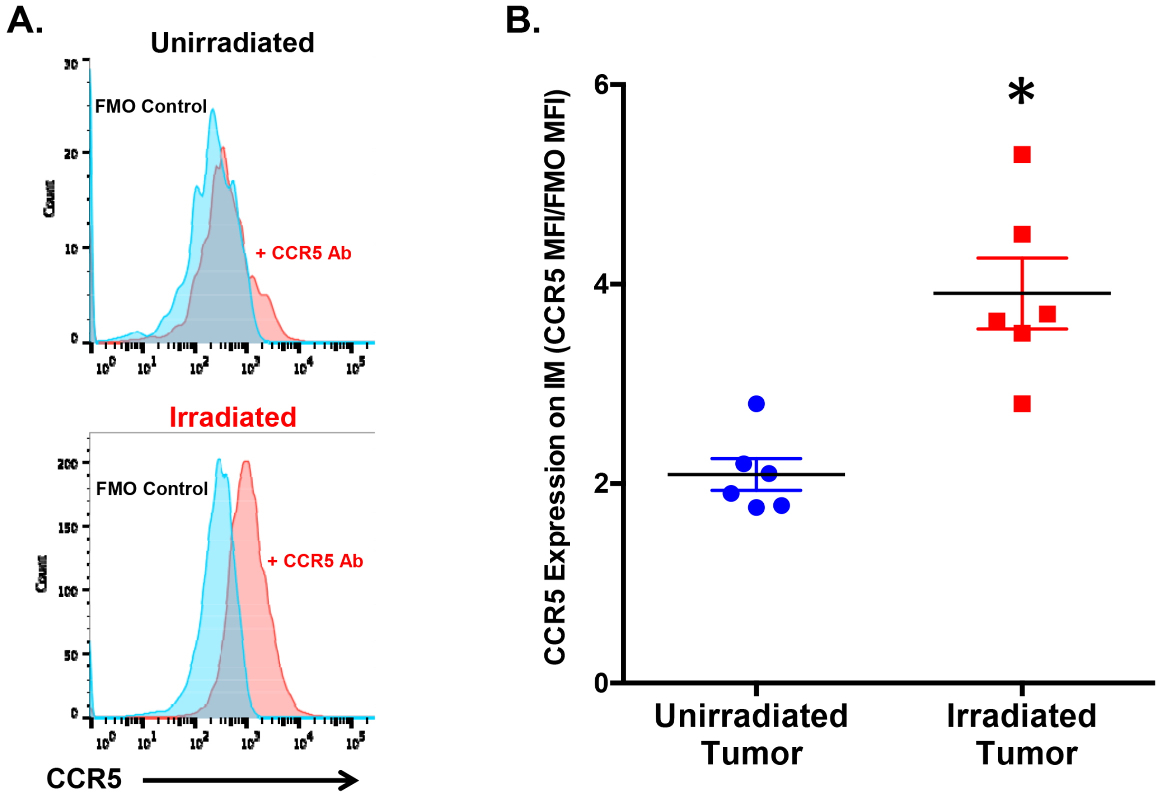

Supplementary Figure S1: Radiotherapy increases the cell surface expression of CCR5 on intratumoral IM. 1x10^5 Colon38 tumor cells were injected i.m. in C57BL/6 mice and left untreated or treated with 15 Gy local radiation on day 7. Four days after RT, tumors were dissociated into a single cell suspension and IM were identified by flow cytometry as described in figure 2. **A.** Representative histograms of CCR5 cell surface expression on intratumoral IM (stained is red whereas FMO control is blue) and quantified in **B.** * represents significance as determined by t-test ($p<0.05$). n=6 per group.

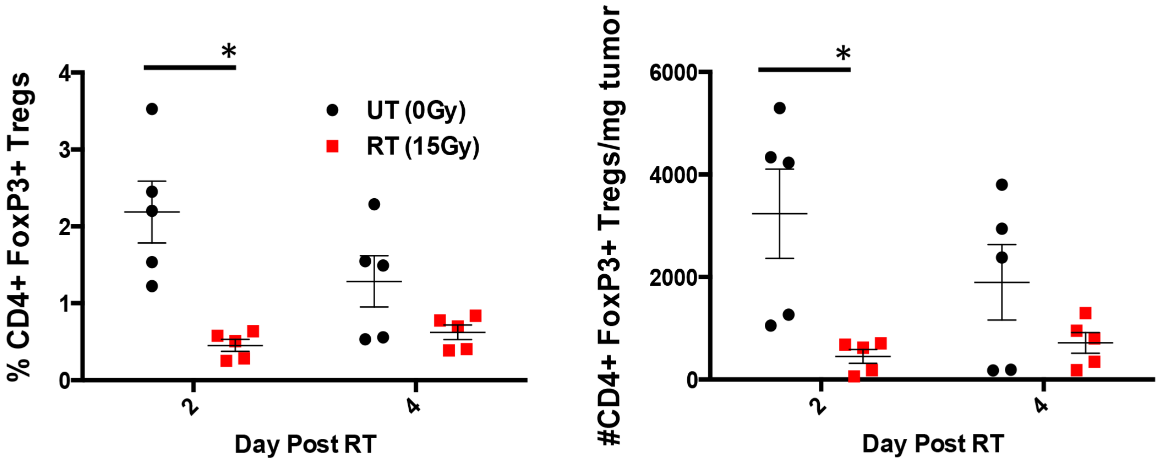

Supplementary Figure S2: CD4+FoxP3+ cells are sparse in Colon38 tumors and are further decreased following RT. $1x10^5$ Colon38 tumor cells were injected i.m. in C57BL/6 mice and left untreated or treated with 15 Gy radiation on day 7 of tumor growth (equivalent to day 0 on x-axis). At 2 or 4 days post RT, tumors were removed, dissociated into a single cell suspension, and examined by flow cytometry for T regulatory (CD45+, CD4+, FoxP3+) cells by percentage of CD45+ cells (left dot plot) and number of cells per mg of tumor (right dot plot). * denotes significance as determined by t-test.

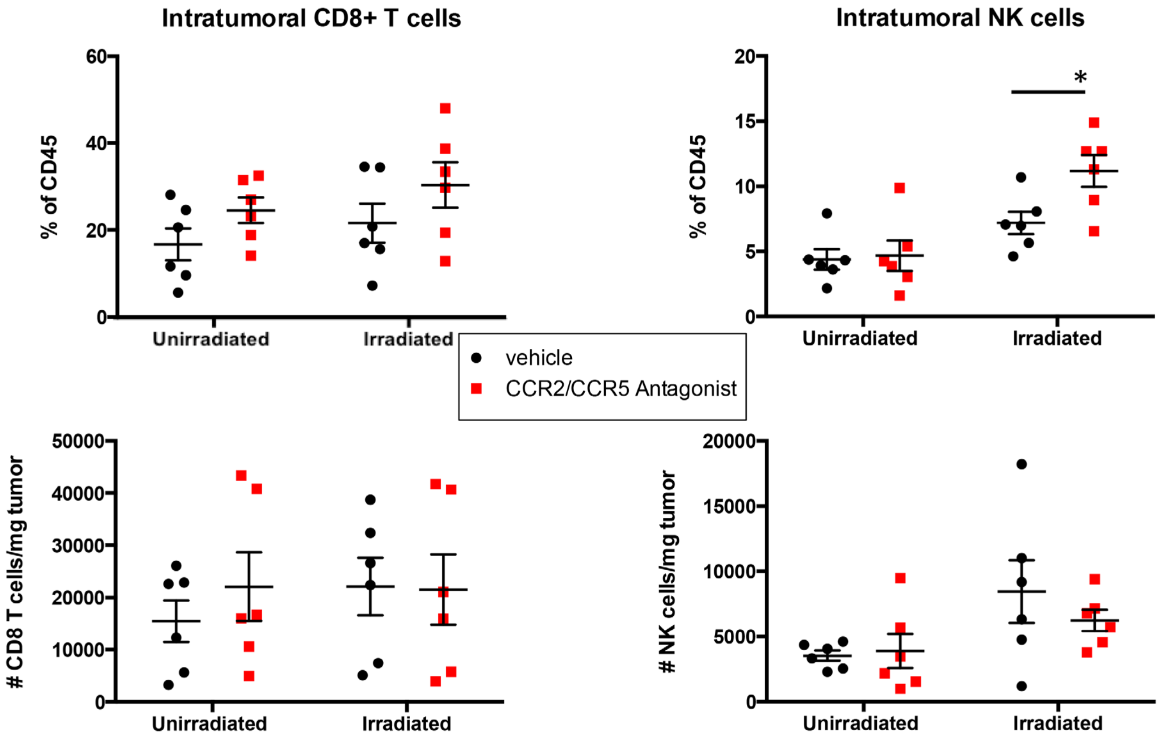

Supplementary Figure S3: CVC does not decrease the intratumoral percentage and number of either NK or CD8+ T cells. Tumors were injected and irradiated as described in Figure 1. Two days previous to irradiation, mice were treated daily with 15 mg/kg drug or vehicle control s.c. for 15 days. Day 11 (day 4 post-RT) tumors were dissociated and intratumoral CD8+ T cells (left side) or NK cells (right side) were quantified by flow cytometry by percentage of total CD45+ immune cells (top panels) and by number of cell/mg tumor tissue (bottom panels). * ($p < 0.05$) represents significance as determined by t-test. n=4-6 for all groups.

Supplementary Table S1: Complete list of genes examined in array plate presented in Figure 3

Target	Regulation	P-Value	Target	Regulation	P-Value
**Adipoq**	-1.17	0.462794	Ifna9	-1.2	0.413095
**Areg**	2.34	0.044467	Ifnab	1.04	0.423521
**Bmp2**	1.23	0.589838	Ifnb1	2.71	0.560087
**Bmp4**	1.1	0.922714	Ifng	2.53	0.025576
**Bmp7**	-1.19	0.406968	Il11	2.38	0.248621
**Ccl11**	5.25	0.001101	Il12a	2.42	0.245723
**Ccl12**	2.18	0.352832	Il15	2.2	0.595048
**Ccl17**	1.73	0.927413	Il17f	1.11	0.653884
**Ccl20**	-1.41	0.42196	Il18	3.52	0.025864
**Ccl21c**	-1.42	0.435691	Il1a	5.42	0.076592
**Ccl22**	1.23	0.629345	Il1b	3.08	0.023445
**Ccl2**	8.40	0.04	Il2	1.79	0.931906
**Ccl4**	2.6	0.016123			
**Ccl5**	4.96	0.03262	Il23a	2.21	0.523344
**Cd40lg**	1.66	0.264432	Il3	-1.07	0.432878
**Cntf**	-1.31	0.391019	Il5	-1.91	0.145047
**Csf1**	1.05	0.871404	Il6	5.38	0.017076
**Csf2**	2.38	0.754439	Il7	-1.33	0.573702
**Csf3**	-1.02	0.456703	Kitl	-1.5	0.246772
**Cx3cl1**	-2.5	0.275721	Lif	1.48	0.42133
**Cxcl1**	2.3	0.018792	Lta	1.15	0.5857
**Cxcl10**	3.6	0.120568	Mif	-1.93	0.02604
**Cxcl12**	2.15	0.241384	Nrg1	1.87	0.220227
**Cxcl3**	3.77	0.593558	Osm	2.32	0.015779
**Cxcl9**	2.5	0.47171	Pf4	2.06	0.082884
**Edn1**	2.18	0.566122	Ppbp	1.76	0.524227
**Fasl**	2.39	0.440722	Sectm1a	2.43	0.027673
**Fgf2**	1.17	0.947463	Slurp1	1.13	0.439835
**Gdf10**	-1.04	0.640941	Spp1	1.34	0.677076
**Gdf15**	2.9	0.064419	Tgfb2	1.17	0.854976
**Gm12597**	-1.87	0.413739	Thpo	1.02	0.517748
**Gm13280**	-1.29	0.414049	Tnf	1.7	0.891292
**Gpi1**	-1.57	0.182924	Tnfrsf11b	2.07	0.145885
**Grn**	2.14	0.196254	Tnfsf10	3.72	0.060232
**Hmgb1**	-1.17	0.379532	Tnfsf11	2.72	0.047416
**Ifna1**	-1.89	0.408337	Tnfsf13b	1.18	0.906575
**Ifna12**	-1.01	0.418662	Vegfa	-1.8	0.221936
**Ifna13**	1.01	0.427012	Wnt1	2.15	0.582489
**Ifna14**	-1.73	0.421865	Wnt5a	-1.19	0.827706
**Ifna2**	-1.25	0.41499			
**Ifna6**	-1.35	0.417126			
**Ifna7**	-1.18	0.396253			

Positive or negative regulation along with P-value (t-test) is included. Genes exhibiting a significant change (irradiated tumors compared to unirradiated tumors) are highlighted in blue.

Supplementary Table S2: Flow cytometry fluorochrome conjugated antibodies

anti-CD4 (GK1.5)	BD Biosciences
NK1.1 (PK136)	BD Biosciences
CD11c (C1-HL3)	BD Biosciences
anti-Ly6C (AL-21)	BD Biosciences
anti-Ly6G (1A8)	BD Biosciences
anti-CD45 (30-F11)	BD Biosciences
anti-CD8 (53-6.7)	eBiosciences
anti-CD11b (M1170)	eBiosciences
F4/80 (1BM8)	eBiosciences
anti-CCR2 (475301)	R&D Systems
anti-CCR5 (7A4)	eBiosciences
